# Interim analysis of postoperative chemoradiotherapy with capecitabine and oxaliplatin versus capecitabine alone for pathological stage II and III rectal cancer: a randomized multicenter phase III trial

**DOI:** 10.18632/oncotarget.8226

**Published:** 2016-03-21

**Authors:** Yan-Ru Feng, Yuan Zhu, Lu-Ying Liu, Wei-Hu Wang, Shu-Lian Wang, Yong-Wen Song, Xin Wang, Yuan Tang, Yue-Ping Liu, Hua Ren, Hui Fang, Shi-Ping Zhang, Xin-Fan Liu, Zi-Hao Yu, Ye-Xiong Li, Jing Jin

**Affiliations:** ^1^ Department of Radiation Oncology, Cancer Hospital, Chinese Academy of Medical Sciences, Peking Union Medical College, Beijing, China; ^2^ Department of Radiation Oncology, Zhejiang Cancer Hospital, Hangzhou, China

**Keywords:** rectal cancer, postoperative chemoradiotherapy, capecitabine, oxaliplatin, phase 3 trial

## Abstract

The aim of this study is to present an interim analysis of a phase III trial (NCT00714077) of postoperative concurrent capecitabine and radiotherapy with or without oxaliplatin for pathological stage II and III rectal cancer. Patients with pathologically confirmed stage II and III rectal cancer were randomized to either radiotherapy with concurrent capecitabine (Cap-RT group) or with capecitabine and oxaliplatin (Capox-RT group). The primary endpoint was 3-year disease-free survival rate (DFS). The 3-year DFS rate was 73.9% in the Capox-RT group and 71.6% in the Cap-RT group (HR 0.92, *p* = 0.647), respectively. No significant difference was observed in overall survival, cumulative incidence of local recurrence and distant metastasis between the two groups (*p* > 0.05). More grade 3–4 acute toxicity was observed in the Capox-RT group than in the Cap-RT group (38.1% vs. 29.2%, *p* = 0.041). Inclusion of oxaliplatin in the capecitabine-based postoperative regimen did not improve DFS but increased toxicities for pathological stage II and III rectal cancer in this interim analysis.

## INTRODUCTION

The optimal sequence and combination of radiotherapy, chemotherapy and surgery for stage II and III rectal cancer have been investigated in several randomized studies. These studies have shown that preoperative chemoradiotherapy was associated with lower treatment-related toxicity, less local recurrence, and improved disease-free survival (DFS) rate when compared with postoperative chemoradiotherapy [1–3]. However, a study with a long-term follow-up revealed no significant differences in the DFS and overall survival (OS) between preoperative and postoperative chemoradiotherapy [4]. Postoperative radiotherapy is still recommended for patients with stages II and III after definitive surgery who did not receive preoperative chemoradiotherapy [5].

In our previous studies [6, 7], the maximum tolerable dose of capecitabine was determined when given concurrently with postoperative radiotherapy, which was 1,600 mg/m^2^ per day administered from days 1–14 with a 7-day rest for two cycles, and the maximum tolerable dose of oxaliplatin combined with 1300 mg/m^2^ capecitabine per day was 80 mg/m^2^. The phase II trial from our center indicated that local recurrence rate was low and distant metastasis was the main treatment failure for pathological stage II and III rectal cancer patients who received capecitabine-based postoperative chemoradiotherapy [8]. Furthermore, inclusion of oxaliplatin in the capecitabine-based postoperative chemoradiotherapy was tolerable [9]. Based on these results [6–9], we designed a phase III trial to see if oxaliplatin incorporated with capecitabine concurrent chemoradiotherapy in postoperative setting could improve 3-year DFS compared with capecitabine alone (registered with ClinicalTrials.gov, number NCT00714077).

## RESULTS

**Figure 1:**
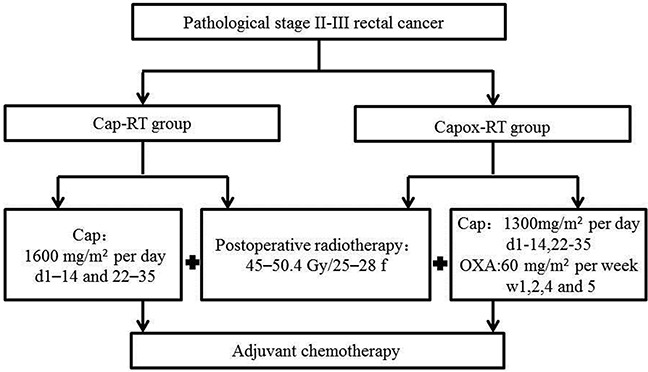
Treatment schedules Cap, capecitabine; OXA, oxaliplatin Capox, capecitabine and oxaliplatin; RT, radiotherapy.

**Figure 2:**
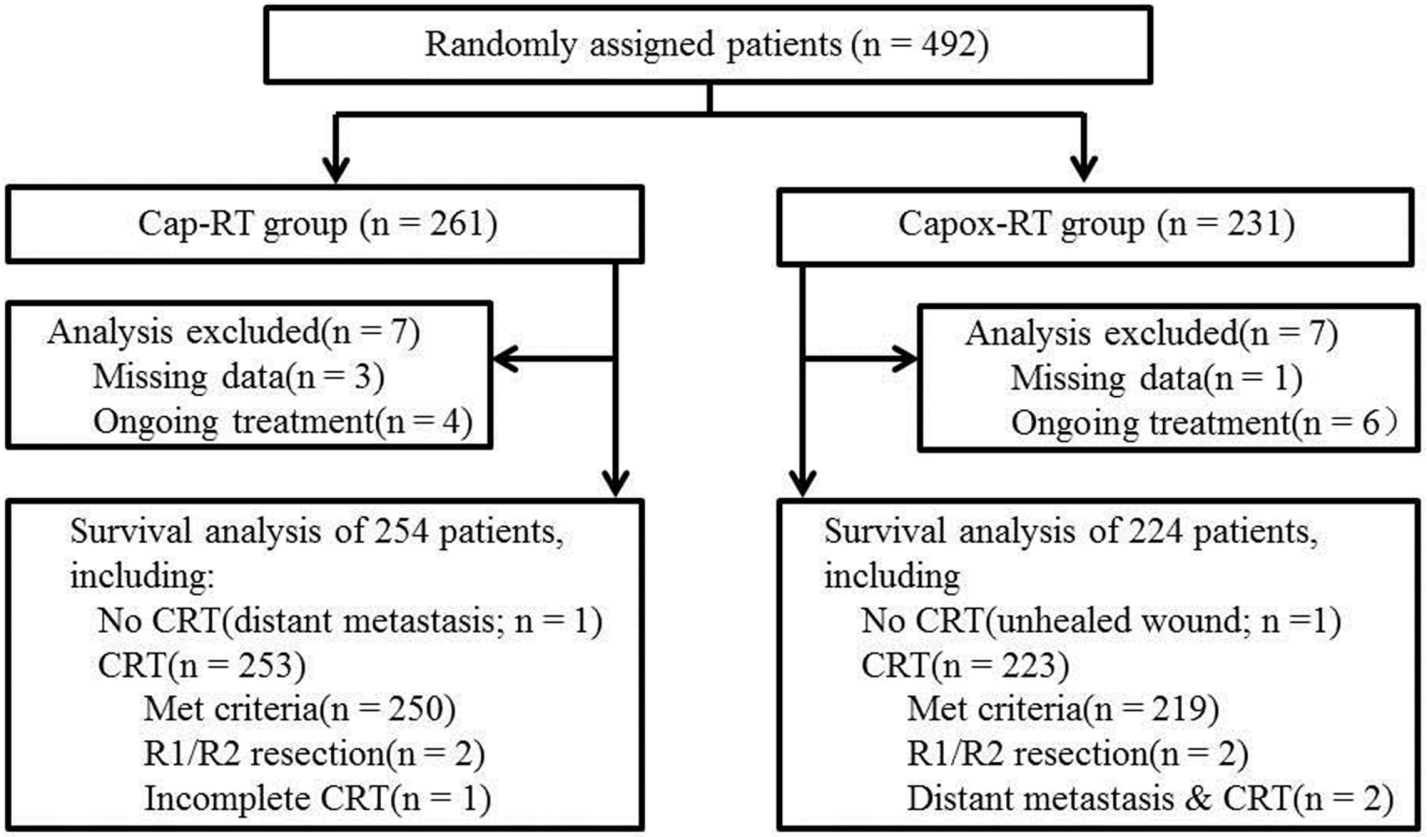
CONSORT diagram CRT, Concurrent chemoradiotherapy; Cap, capecitabine; Capox, capecitabine and oxaliplatin; RT, radiotherapy.

Between April 1, 2008 and July 30, 2014, we enrolled 492 participants from three centers in China. Of these participants recruited, 478 were evaluable (254 in the Cap-RT group and 224 in the Capox-RT group, Figure 1 and 2). Baseline characteristics were well balanced between the two groups (Table 1).

**Table 1:** Baseline characteristics

Characteristics	Cap-RT *N* = 254 (%)	Capox-RT *N* = 224 (%)	*P*
Gender			
Male	166 (65.4)	141 (62.9)	0.584
Female	88 (34.6)	83 (37.1)	
Age (years)			
Median	55	55	0.681
Range	24-73	19-75	
Distance from anal verge (cm)			
≤5	113(44.5)	84 (37.5)	0.248
5.1-10	98(38.6)	102(45.5)	
>10	43(16.9)	38(17.0)	
KPS			
≥80	248(97.6)	212(94.6)	0.086
<80	6(2.4)	12(5.4)	
pT classification			
T2	22(8.7)	21(9.4)	0.169
T3	220(86.6)	183(81.7)	
T4	12(4.8)	20(9.0)	
pN classification			
N0	65(25.6)	52(23.2)	0.819
N1	115(45.3)	103(46.0)	
N2	74(29.1)	69(30.8)	
TNM stage			
II	65(25.6)	52(23.2)	0.686
III	189(74.4)	172(76.8)	
Surgery			
Anterior resection	190(74.8)	169(75.4)	0.871
Abdominoperineal resection	64(25.2)	55(24.6)	
Lymphovascular invasion			
No	207(81.5)	182(81.2)	0.945
Yes	47(18.5)	42(18.8)	
Tumor deposits			
No	212(83.5)	190(84.8)	0.686
Yes	42(16.5)	34(15.2)	
Number of nodes retrieved			
Median	17	17	0.952
Range	2-72	2-51	
Number of positive nodes			
Median	2	2	0.824
Range	0-24	0-28	
Techniques*			
Conventional RT	6(2.4)	6(2.7)	0.566
3D-CRT	54(21.3)	39(17.5)	
IMRT	193(76.3)	178(79.8)	

Abbreviations: KPS, Karnofsky performance status; RT, radiotherapy; 3D-CRT, 3 dimensional conformal radiotherapy; IMRT, intensity modulated radiotherapy; Cap, capecitabine; Capox, capecitabine and oxaliplatin. Data represent number of patients (%) unless otherwise stated.

* 253 patients in the Cap-RT group and 223 patients Capox-RT group received chemoradiotherapy.

### Disease-free and overall survival

**Figure 3:**
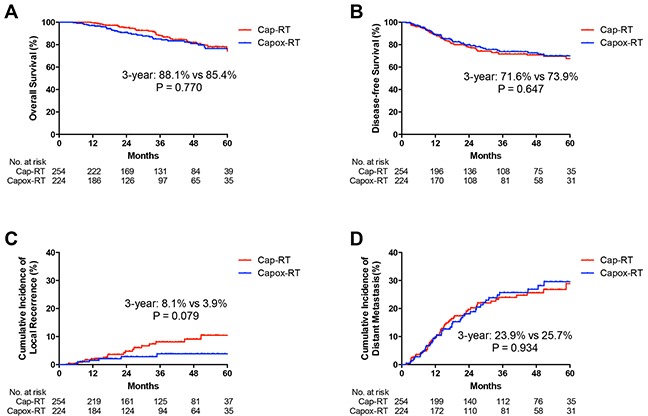
Kaplan-Meier curves of A. overall survival (OS), B. disease-free survival (DFS), C. cumulative incidence of local recurrence and D. cumulative incidence of distant metastasis for in Cap-RT and Capox-RT groups Cap, capecitabine; Capox, capecitabine and oxaliplatin; RT, radiotherapy.

**Figure 4:**
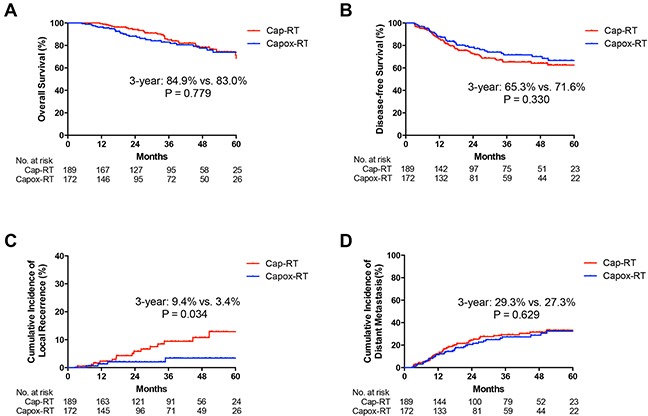
Kaplan-Meier curves of A. overall survival (OS), B. disease-free survival (DFS), C. cumulative incidence of local recurrence and D. cumulative incidence of distant metastasis for stage III patients in Cap-RT and Capox-RT groups Cap, capecitabine; Capox, capecitabine and oxaliplatin; RT, radiotherapy.

The 3-year DFS rates were 73.9% and 71.6% in the Capox-RT and Cap-RT groups (HR 0.92, 95% CI: 0.63–1.34, *p* = 0.647), respectively. No statistically significant difference was observed in the OS between the two groups (*p* > 0.05) (Figure 3A-3B). No statistically significant differences were observed in OS and DFS for patients with stage II or III between the two groups (*p* > 0.05) (Figure 4A-4B).

### Local recurrence and distant metastasis

No statistically significant difference was observed in the incidence of local recurrence and distant metastasis between the two groups (3-year cumulative incidence of local recurrence: 3.9% vs. 8.1%, HR 0.44, 95% CI: 0.18–1.13, *p* = 0.079; distant metastasis: 25.7% vs. 23.9%, HR 1.02, 95% CI: 0.69–1.51, *p* = 0.934 in the Capox-RT and Cap-RT groups, respectively; Figure 3C-3D). For stage III patients, the 3-year cumulative incidence of local recurrence was significantly lower in the Capox-RT group (3.4% vs. 9.4%, *p* = 0.034, Figure 4C). No benefits were observed in terms of the 3-year cumulative incidence of distant metastasis for patients with stage III rectal cancer in the Capox-RT group (Figure 4D).

### Compliance

In the Cap-RT group, 94.1% (238/253) and 92.1% (233/253) patients completed radiotherapy and concurrent chemotherapy on schedule, respectively. However, in the Capox-RT group, only 85.7% (191/223) and 74.4% (166/223) patients did. The number of patients who had to stop or interrupt radiotherapy due to grade 3-4 toxicity in the Cap-RT and Capox-RT groups was 14 and 27, respectively. Nineteen patients had to modify their concurrent chemotherapy due to grade 3-4 toxicity, whereas, there were 51 patients in the Capox-RT group due to the same situation. There was significant difference between these two groups in the completion of both radiotherapy and concurrent chemotherapy (*p* < 0.05). The percentages of patients who received a <45 Gy dose in the Capox-RT and Cap-RT groups were 4.0% and 2.4%, respectively (*p* = 0.022). The percentages of patients who received ≥75% of the full dose of concurrent chemotherapy in the Capox-RT and Cap-RT groups were 94.2% and 97.2%, respectively (*p* = 0.097).

### Adjuvant chemotherapy

A total of 193 (76.0%) patients in the Cap-RT group and 151 (67.4%) in the Capox-RT group received adjuvant chemotherapy (*p* = 0.055). The reasons of 113 patients with no adjuvant chemotherapy were described as follows: 22 patients refused; 8 patients with disease progression during chemoradiation or before the start of adjuvant chemotherapy; 3 patients with poor blood condition and 80 patients unknown. Another 21 patients completed chemoradiation and did not start adjuvant chemotherapy at the time of analysis.

### Acute toxicities

There was no significant difference between the two groups at any grade of acute toxicities, however, grades 3–4 acute toxicities were observed in 38.1% and 29.2% of patients in the Capox-RT and Cap-RT groups, respectively (*p* = 0.041). The details of acute toxicity data are reported in Table 2.

**Table 2:** Acute toxicity in patients who received postoperative chemoradiotherapy

Acute Toxicity	All Grades		*P*	Grade 3-4		*P*
	Cap-RT *N*=253	Capox-RT *N*=223		Cap-RT *N*=253	Capox-RT *N*=223	
All	247(97.6)	221(99.1)	0.293	73(28.9)	84(37.7)	0.041
GI toxicity						
Anorexia	126(49.8)	145(65.0)	0.001	1(0.4)	4(1.8)	0.149
Nausea	77(30.4)	123(55.2)	<0.001	0(0.0)	5(2.2)	0.022
Vomiting	23(9.1)	43(19.3)	0.001	0(0.0)	4(1.8)	0.047
Diarrhea/Proctitis	170(67.2)	164(73.5)	0.131	54(21.3)	61(27.4)	0.126
Tenesmus	145(57.3)	145(65.0)	0.085	5(2.0)	12(5.4)	0.046
Blood toxicity						
Leucopenia	189(74.7)	156(70.0)	0.247	8(3.2)	7(3.1)	0.989
Thrombocytopenia	16(6.3)	30(13.5)	0.009	0(0.0)	1(0.4)	0.468
ALT/Bil elevation	6(2.4)	15(6.7)	0.021	0(0.0)	0(0.0)	-
Others						
Body weight loss	18(7.1)	19(8.5)	0.568	0(0.0)	0(0.0)	-
Fatigue	146(57.7)	153(68.6)	0.014	1(0.4)	7(3.1)	0.029
Radiation dermatitis	164(64.8)	146(65.5)	0.882	13(5.1)	6(2.7)	0.173
Neuropathy	5(2.0)	28(12.6)	<0.001	0(0.0)	0(0.0)	-
Hand-foot syndrome	15(5.9)	19(8.5)	0.273	0(0.0)	0(0.0)	-

Abbreviations: Cap, capecitabine; Capox, capecitabine and oxaliplatin; RT, radiotherapy; GI, gastrointestinal. Data represent number of patients (%).

## DISCUSSION

With the publication of the clinical trials (ACCORD, NSABP R-04, CAO/ARO/AIO-04, STAR-01 and PETACC-6) [10–14], the poor patient accrual and the median follow-up of ~3 years for alive patients in this trial, we present this interim analysis to evaluate whether the results of our study are similar to the previous reports [10–13]. This interim analysis revealed no statistically significant difference in the DFS between the two groups and the addition of oxaliplatin increased toxicities, which was similar to the findings of the ACCORD, NSABP R-04, and PETACC-6 studies [10–12]. To our knowledge, this is the only study to investigate the role of oxaliplatin plus postoperative capecitabine-based chemoradiation in stage II and III rectal cancer.

A German trial by Hofheinz et al [15] investigated the efficacy and safety of substituting fluorouracil with capecitabine for perioperative treatment of locally advanced rectal cancer (LARC). The results of this trial revealed that the 5-year OS in the capecitabine group was non-inferior to that in the fluorouracil group and capecitabine could replace fluorouracil in adjuvant or neoadjuvant chemoradiotherapy for LARC. It was noticed that fewer patients developed distant metastases in the capecitabine group (19% vs. 28%, *p* = 0.04). Interestingly, the CAO/ARO/AIO-04 trial [14] indicated that addition of oxaliplatin to fluorouracil-based regimens could improve the 3-year DFS (71.2% vs. 75.9%, *p* = 0.03) for LARC. The results of our present study and those published trials [10–12] in which preoperative concurrent chemoradiotherapy was investigated suggested that adding a weekly dose of oxaliplatin to capecitabine-based chemoradiotherapy regimens did not improve the 3-year DFS. One possible reason is that capecitabine might be superior to 5-fluorouracil and offsets the efficiency of oxaliplatin on DFS.

The ADORE trial [16] and the CAO/ARO/AIO-04 trial [14] showed a benefit of adjuvant oxaliplatin-based chemotherapy for rectal cancer. In the current study, adjuvant chemotherapy was administered in 76% of patients treated with Cap-RT and in 67% of patients treated with Capox-RT (*p* = 0.055); most of the patients received XELOX or FOLFOX as adjuvant chemotherapy regimens. This may also mask the efficacy of oxaliplatin in concurrent chemoradiation.

The 3-year cumulative local recurrence rate in our study was 8.1% in the Cap-RT group and 3.9% in the Capox-RT group, which is similar to the results of PETACC-6 (7.6% and 4.6%, respectively) [12]. The incidence of local recurrence for patients with pathological stage III cancer was lower in the Capox-RT group (9.4% vs 3.4 %, *p* = 0.034). The same excellent local control was also observed in other studies conducted by our center [17, 18]. We have to notice that the lower local recurrence for patients with pathological stage III rectal cancer in the Capox-RT group should be interpreted with caution as it was the results of interim analysis. The 3-year cumulative incidence of distant metastasis was 23.9-25.7%, similar to the results of ACCORD (22–24%) and PETACC-6 (17.6–19.2%) [10, 12], which was the main factor affecting the DFS and OS. Unfortunately, adding oxaliplatin to the concurrent regimen did not prevent distant metastasis, regardless of preoperative or postoperative settings.

Previous randomized trials of preoperative chemoradiation showed that grade 3-4 toxicities occurred in 6.6–15.1% of patients receiving capecitabine and in 15.4–36.7% of patients receiving capecitabine and oxaliplatin [19–21]. In the present study, grade 3–4 acute toxicities were higher than those receiving preoperative chemoradiation, especially in the Capox-RT group (38.1% vs 29.2%). Even though the majority patients in the Capox-RT group were able to complete the designed adjuvant chemoradiotherapy (the rates of receiving a dose of lower than 45 Gy were less than 5.0% and more than 94% patients received ≥75% of full dose of concurrent chemotherapy), adding oxaliplatin to concurrent chemoradiation increased toxicities with no benefits of DFS.

The limitations of the study should be acknowledged. First, concurrent chemoradiation after surgery for pathological stages II and III rectal cancer was the main treatment modality in China before 2008. That was the reason that we carried the study based on postoperative chemoradiation rather than preoperative chemoradiation. Since the publication of the clinical trial [1] in German, preoperative chemoradiotherapy has become a priority. This change has led to difficulty in accruing patients for the current study, especially after the year of 2010. However, postoperative chemoradiation is still recommended for patients with stages II and III rectal cancer after definitive surgery who did not receive preoperative chemoradiotherapy [5]. Second, this is not a pre-planned interim analysis, under-powered, and the follow-up is short. By the end of 2015, the patients’ enrollment is going to be finished and the final result is anticipated.

## MATERIALS AND METHODS

### Study design and patients

This is a multicenter, open-label, randomized, phase III trial. The protocol was approved by the Independent Ethics Committee of all participating institutions. Each patient provided written informed consent before participation. Eligible patients were 18–75 years old, who had a R0 total mesorectal excision (TME) with pathological stage II or III rectal adenocarcinoma (regardless of clinical stage before surgery). The upper border of the tumor was below L5. Further inclusion criteria were Karnofsky performance status (KPS) of 70 or higher and adequate hematological, liver, and renal function. Exclusion criteria included metastatic disease, prior radiotherapy or chemotherapy, presence of cancers other than basal cell carcinoma of the skin or carcinoma in situ of the uterine cervix, pregnancy, lactation, any concomitant illness potentially affecting treatment compliance, and known peripheral neuropathy.

### Randomization

Patients who met the criteria were enrolled and randomized (1:1) into one of two groups (Figure 1): the control group (Cap-RT group: Patients received postoperative concurrent chemoradiotherapy with capecitabine), and the experimental group (Capox-RT group: Patients received postoperative concurrent chemoradiotherapy with capecitabine plus oxaliplatin). Randomization was performed centrally at the study administration office according to a computer-generated randomization codes with stratification of pathological stage (II vs. III). The treatment groups were not masked throughout the trial because the treatments involved different administration and schedules.

### Procedures

Radiotherapy consisted of 45 Gy in 25 fractions of 1.8 Gy, 50.4 Gy in 28 fractions of 1.8 Gy or 50 Gy in 25 fractions of 2.0 Gy (6 MV photons), five times per week, over 5–5.5 weeks using three-field two-dimentional conventional radiotherapy (2D-RT), three-dimensional conformal radiotherapy (3D-CRT) or intensity-modulated radiotherapy (IMRT) technique. The clinical target volume (CTV) was delineated according to Roels’ guidelines [22], the same as studies described previously [6–8].

In the Cap-RT group, concurrent chemotherapy consisted of two cycles of oral capecitabine (1,600 mg/m^2^) on days 1–14 and 22–35. The Capox-RT group received the same postoperative radiotherapy as the Cap-RT group, combined with oral capecitabine (1,300 mg/m^2^) on days 1–14 and 22–35, and a 2-h infusion of oxaliplatin (60 mg/m^2^) on weeks 1, 2, 4, and 5. The dose and usage of capecitabine and oxaliplatin refered to the previous studies [6, 7]. Following 4 weeks of completing chemoradiation, 4~6 cycles of XELOX (capecitabine and oxaliplatin) or 8~12 cycles of FOLFOX (fluorouracil, leucovorin, and oxaliplatin) was delivered.

Vital signs, chemistry panel and complete blood count were monitored weekly during chemoradiotherapy and before each adjuvant chemotherapy cycle. The acute toxicity of postoperative concurrent chemoradiotherapy was scored according to the Common Terminology Criteria for Adverse Events (CTCAE v. 3.0). Oxaliplatin was interrupted if a grade 3 or 4 toxicity was encountered, and capecitabine was continued. When the severity of the toxicity had decreased to grade 0 or 1 after appropriate symptomatic relieving therapy, oxaliplatin was restarted at 75% and 50% of the original dose at the first and second appearance of the respective toxicity, respectively. Capecitabine was withdrawn in the following situations: ≥grade 2 hand-foot syndrome and persistent grade 2 toxicities after the second oxaliplatin dose reduction. Radiotherapy was not modified except when unrecoverable grade 4 toxicities developed. In that case, both chemotherapy and radiotherapy were discontinued [6].

Baseline assessments included medical history, physical examination, liver and renal function test, complete blood count, abdominal ultrasound and/or computed tomography (CT), pelvic CT and chest radiograph after surgery. Follow-up assessments included physical examination, liver and renal function test, complete blood count and tumor markers [carcinoembryonic antigen (CEA) and cancer antigen 19-9 (CA19-9)] every 3 months for the first 2 years, and every 6 months thereafter. Abdominal ultrasound and/or CT, pelvic CT or MRI, and chest radiograph were performed every 6 months. Colonoscopic examination was repeated annually.

### Statistical analysis

Statistical tests were based on a two-sided significance level. A *p* value of < 0.05 was considered statistically significant. The primary endpoint was 3y-DFS. Assuming a 10% dropout rate, a total of 570 patients (285 per group) provided 80% power to detect 3-year DFS of 65% for the Cap-RT group and 75% for the Capox-RT group, with a two-sided α of 0.05. Secondary endpoints included OS, cumulative incidence of local recurrence, cumulative incidence of distant metastasis, compliance and safety. Safety and compliance were evaluated in the per-protocol (PP) analysis excluding the ineligible patients, and efficacy endpoints were analyzed according to the intention-to-treat principle. Statistical analysis was carried out using SPSS version 22.0 (Chicago, IL). The cumulative incidence of local recurrence, cumulative incidence of distant metastasis, DFS and OS were defined as the day of surgery to the date of the event and were estimated with the Kaplan-Meier method. The log-rank test was used to assess differences in time-to-event outcomes. Chi-square and Mann-Whitney U tests were used to compare the differences of categorical variables and continuous variables between the Cap-RT and the Capox-RT group, respectively.

As the results of the clinical trials (ACCORD, NSABP R-04, CAO/ARO/AIO-04, STAR-01 and PETACC-6) [10–14] had been released recently, most of them showed no benefits of oxaliplatin when combined with capecitabine or fluorouracil in preoperative chemoradiotherapy setting. After discussion with the investigators of other centers, we decided to perform an earlier analysis than the final.

## CONCLUSION

Inclusion of oxaliplatin in the capecitabine-based postoperative regimen did not improve DFS but increased toxicities for pathological stage II and III rectal cancer in this interim analysis.
